# Outcomes in Advanced Stage Epithelial Ovarian, Fallopian Tubal, and Peritoneal Cancer after Primary Surgery and Adjuvant Chemotherapies: A Single-Institute Real-World Experience

**DOI:** 10.3390/ijerph17103523

**Published:** 2020-05-18

**Authors:** Chia-Hua Chang, Hsiao-Li Kuo, Tzu-Chien Chen, Chia-Sui Weng, Ling Lim, Wan-Chun Huang, Chih-Long Chang, Tsung-Hsien Su, Kuo-Gon Wang, Kung-Liahng Wang, Yuh-Cheng Yang, Jen-Ruei Chen

**Affiliations:** 1 Department of Obstetric & Gynecology, MacKay Memorial Hospital, Taipei 10449, Taiwan; trumpet_1323@hotmail.com (C.-H.C.); chen.4418@mmh.org.tw (T.-C.C.); jenny6939@mmh.org.tw (C.-S.W.); 4442.4442@mmh.org.tw (L.L.); 4445.4445@mmh.org.tw (W.-C.H.); ccl@mmh.org.tw (C.-L.C.); aikuo7@mmh.org.tw (K.-G.W.); eugene@mmh.org.tw (Y.-C.Y.); 2 Department of Nursing, MacKay Memorial Hospital, Taipei 10449, Taiwan; rl892081.d786@mmh.org.tw; 3 Department of Obstetric & Gynecology, MacKay Memorial Hospital, Hsinchu Branch, Hsinchu 30071, Taiwan; sutsung@mmh.org.tw; 4 Department of Obstetric & Gynecology, MacKay Memorial Hospital, Taitung Branch, Taitung 95054, Taiwan; klwang@mmh.org.tw; 5 Department of General Education, MacKay Junior College of Medicine, Nursing and Management, New Taipei City 11260, Taiwan

**Keywords:** fallopian tubal cancer, chemotherapy, lymphadenectomy, ovarian cancer, peritoneal cancer

## Abstract

Debulking surgery followed by systemic chemotherapy—including three-weekly intravenous paclitaxel and carboplatin (GOG-158)—is the cornerstone for advanced epithelial ovarian, fallopian tubal, and peritoneal cancer (EOC) treatment. In this scenario, Federation of Gynecology and Obstetrics (FIGO) stage, cell types, completeness of surgery, lymph nodes (LN) status, adjuvant chemotherapy regimens, survival status, progression-free survival (PFS), and overall survival (OS) of 192 patients diagnosed as having stage IIIA1–IVB EOC over January 2008–December 2017 were analyzed retrospectively. Of them, 100 (52.1%) patients had been debulked optimally. Of all cases, 64.1% and 10.9% demonstrated serous and clear-cell carcinoma. Moreover, the FIGO stage, surgery completeness, and LN status affected recurrence/persistence and mortality (all *p* < 0.001). Clear cell carcinoma led to shorter survival than serous carcinoma (*p* = 0.002). Adjuvant chemotherapy regimens were divided into five main groups according to previous clinical trials. However, choice of chemotherapy failed to demonstrate significant differences in patient outcomes. Similar results were found in the sub-analysis of optimally debulked cases, except that intraperitoneal chemotherapy could reduce mortality risk when compared with GOG-158 (*p* = 0.042). Notably, retroperitoneal LN dissection in all cases or optimally debulked cases reduced risks of recurrence/persistence and mortality, and prolonged PFS and OS significantly (all *p* < 0.05). Without optimal debulking, LN dissection led to little improvement in outcomes. Various modified chemotherapy regimens did not prolong PFS and OS or reduce recurrence/persistence and mortality risks. LN dissection is strongly recommended to improve the completeness of surgery and patient outcome. Clear cell type has a poorer outcome than serous type, which requires more aggressive treatment and follow-up.

## 1. Introduction

Ovarian cancer is the second and third most common gynecologic malignancy in developed and developing countries, respectively [1]. The most common histological type is epithelial [2]. Because of its non-specific symptoms and non-useful screening methods, approximately 70–75% cases are at advanced stages at the time of diagnosis. Advanced epithelial ovarian, fallopian tubal, and peritoneal cancer (EOC) is characterized by a high recurrence rate within two years after primary management and a low five-year survival rate [1].

Currently, the Federation of Gynecology and Obstetrics (FIGO) revised the staging system of EOC since 2014 [3]. In early stage, stage IC was separated into three sub-categories and stage IIC was removed. In advanced disease, solitary retroperitoneal lympho nodes (LN) metastases without peritoneal disease was re-staged as IIIA1. Retroperitoneal spread of EOC seems to have a better prognosis than intraperitoneal tumor dissemination. Occasionally, retroperitoneal LN dissection would not be performed if peritoneal carcinomatosis is encountered during a primary or interval debulking operation, which are cases with major medical problems or unstable hemodynamic condition during surgery.

Primary debulking surgery followed by systemic chemotherapy is the preferred treatment for advanced EOC. Advancements in systemic therapies, including chemotherapy and anti-vascular target therapy, have considerably improved the outcomes of patients with advanced EOC. The most promising results have come from the Gynecologic Oncology Group (GOG) trials in the United States, which led to increased progression-free survival (PFS) and overall survival (OS) since 1996. Addition of paclitaxel in platinum-based regimens was found to lead to improved outcomes compared with the traditional regimen of cyclophosphamide plus cisplatin [4]. A 3-h infusion of paclitaxel plus carboplatin had equal PFS but less comorbidity than a 24-h infusion of paclitaxel plus cisplatin in GOG-158 in 2003 [5]. Intraperitoneal chemotherapy with paclitaxel and cisplatin in optimally debulked patients with EOC had promising PFS in a phase-3 trial of GOG-175 in 2006 [6]. Schedule modification of weekly infusion of paclitaxel plus three-week carboplatin was studied in Japan’s GOG (JGOG-3016), which revealed substantial improvement in PFS and OS when compared with other trials [7]. Adding anti-vascular bevacizumab (Genentech, South San Francisco, CA, USA) in the traditional three-weekly paclitaxel–carboplatin chemotherapy plus maintenance also led to better PFS in GOG-218 and ICON-7 [8,9]. Nevertheless, determination of the most effective chemotherapy regimen is challenging because of a lack of more substantial randomized trials and the unfeasibility of a direct cross-trial comparison.

In this study, with an aim to provide useful data for selecting the best treatment approach in real-world practice, we, retrospectively, evaluated the factors that could interfere with outcomes of patients with stage III and IV EOC.

## 2. Materials and Methods

### 2.1. Patients and Definitions

After obtaining Institutional Review Board approval (approval No. 20MMHIS023e) from our institute, we, retrospectively, reviewed medical records of 788 patients diagnosed as having EOC between 1 January 2008 and 31 December 2017. We included only patients with stage III or IV EOC, staged as per the 2014 revision of the International Federation of Gynecology and Obstetrics (FIGO) staging system. Both primary staging or debulking surgery as well as the subsequent adjuvant chemotherapy were completed in the same medical center (MacKay Memorial Hospital, Taipei, Taiwan). All of them had at least two years of follow-up. If the primary surgery was not performed at our institute, patients were included only when operative records and pathology slides were available. Among patients with non-EOC histology, no available review of surgery or pathology, concurrent secondary malignancy, or incomplete courses of chemotherapy were excluded. Lastly, 192 patients who met all the inclusion criteria were included.

Patients’ basic characteristics, such as FIGO stages, cell types, surgical completeness, lymph nodes (LN) status (metastasis), chemotherapy regimens (five major adjuvant systemic chemotherapy regimens), first recurrent status, survival status, PFS, and OS are listed in Table 1. Adjuvant chemotherapy regimens were summarized into five groups according to their clinical trials in the literature in Table 2. Patients’ primary regimen of chemotherapy before interval debulking surgery was omitted in the grouping if neoadjuvant chemotherapy was administered. PFS was defined as the time from surgery to first imaging-confirmed recurrence based on response evaluation criteria of the solid tumor (RECIST) 1.1 version. Patients who were refractory to platinum-based regimens were considered as having 0 months of PFS. OS was defined as the time from surgery to death or last visit at our clinic.

To eliminate the bias of outcome from different surgical completeness, cases who achieved optimal debulking (n = 100) were selected for further analysis.

### 2.2. Statistical Analysis

All statistical analyses were performed with SPSS (version 21.0, IBM Corp., Armonk, NY, USA) and R (R Core Team, 2019, Vienna, Austria) [11]. Continuous variables were compared with Student’s t-test or Wilcoxon–Mann–Whitney test according to data distribution. Categorical and continuous variables were compared using the chi-square test, Fisher’s exact test, and the Mann–Whitney U test, respectively. We used the Cox regression hazard model to calculate hazard ratios (HRs) of recurrence/persistence and mortality inside each factor. PFS and OS were evaluated using the Kaplan–Meier method and compared using the log-rank test. *p* value < 0.05 was considered statistically significant. All survival curves were produced using the package of “survminer” from R language [12].

## 3. Results

In total, 192 patients were included in this study (median age, 53 years, range 29–81 years). Of them, most (n = 123, 64.1%) cases had serous carcinoma followed by clear cell carcinoma (n = 20, 10.9%) cases. More than half of the cases (n = 100, 52.1%) completed the optimal debulking surgery, and the remaining cases received suboptimal (n = 75, 39.1%) or no debulking surgery (biopsy and cytology proof only, n = 17, 8.9%). Most cases were of FIGO stage IIIC (n = 100, 52.1%), and most patients (n = 144, 75%) received the traditional regimen of three-weekly intravenous infusion with paclitaxel 175 mg/m^2^ plus a carboplatin area under the curve (AUC) 5 for at least six courses. Patients’ clinical and demographic characteristics are summarized in Table 1.

**Figure 1:**
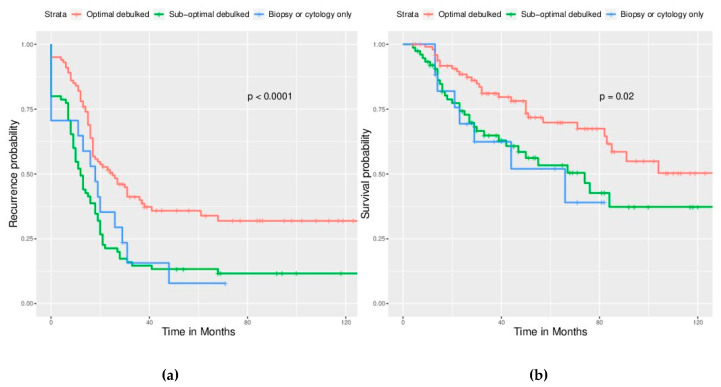
Among survival curves by surgical completeness, significantly better survival can be observed in the optimally debulked group. (**a**) Progression free survival (PFS), *p* < 0.0001. (**b**) Overall survival (OS), *p* = 0.02. However, no significant survival difference between the sub-optimal debulked group and non-debulked group. (*p* is not shown).

Primary debulking surgery is the cornerstone in the primary treatment of EOC. We found that optimal debulking surgery led to better outcomes—significantly longer PFS (22 ± 32.07 months) than suboptimal debulking (12 ± 30.18 months) and no debulking (18 ± 19.12 months, *p* < 0.001) as well as significantly longer OS (47.5 ± 33.03 months vs. 33 ± 32.68 months and 29 ± 24.40 months, *p* < 0.001) (Table 3). Suboptimal debulking led to PFS and OS identical to no debulking. Survival curves were demonstrated in Figure 1a,b.

### 3.1. Analysis of All Enrolled Cases (n = 192)

#### 3.1.1. Factors Affecting Disease Recurrence/Persistence and Survival

In all enrolled cases, FIGO stage (*p* = 0.001), surgical completeness (*p* < 0.001), and LN status (*p* < 0.001) were significant factors affecting disease recurrence/persistence, but cell types (*p* = 0.444) and adjuvant chemotherapies (*p* = 0.10) were not (Table 4). Furthermore, surgical completeness (*p* = 0.004) and LN status (*p* < 0.001) significantly affected survival, whereas the FIGO stage (*p* = 0.062), cell types (*p* = 0.333), and adjuvant chemotherapy (*p* = 0.244) did not (Table 5). Notably, choice of adjuvant chemotherapy played little role in affecting recurrence/persistence and survival.

#### 3.1.2. Risk Assessment

We used the Cox regression hazards model to analyze the relative risk of each factor for recurrence/persistence and survival status (Table 6 and Table 7). Risk of disease recurrence/persistence increased with the FIGO stage, and the FIGO stage ≥ IVA increased the risk of mortality. Clear cell type was found to have a higher risk of disease recurrence/persistence (Hazard Ratio (HR) = 2.453, 95% Confident Interval (CI) = 1.377–4.365, *p* = 0.002) and mortality (HR = 6.278, 95% CI = 2.945–13.432, *p* < 0.001) than serous type. The endometrioid type (HR = 4.695, 95% CI = 1.620–13.610, *p* = 0.004) also showed a higher mortality risk than serous type. In cases whose LN dissection was carried out during surgery, the risks of disease recurrence/persistence and mortality were lower than in cases without LN dissection. In risk of recurrence/persistence, when compared with LN dissection with positive nodes to no LN dissection, HR is 0.528 (95% CI = 0.331–0.845, *p* = 0.008). Additionally, comparing LN dissection with negative nodes to no LN dissection, HR is 0.331 (95% CI = 0.162–0.585, *p* < 0.001). In risk of mortality, it shows that LN dissection actually decreased the risk of mortality. (For more details, please refer to Table 7). Lastly, none of the modified chemotherapy regimens demonstrated a higher or lower risk of disease recurrence/persistence and mortality than the traditional three-weekly intravenous paclitaxel + carboplatin regimen from GOG-158.

#### 3.1.3. Survival Analysis

**Figure 2:**
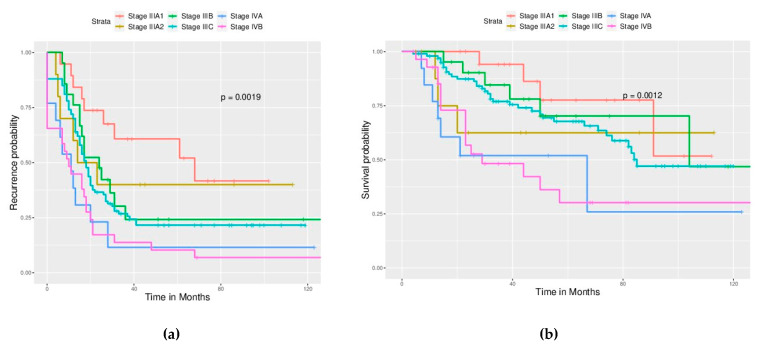
Survival curves based on the FIGO stage (All enrolled cases) (**a**) PFS, *p* = 0.0019. (**b**) OS, *p* = 0.0012. Stage IIIA1 has better PFS than other stages, and stage IV has worse OS than stage III.

**Figure 3:**
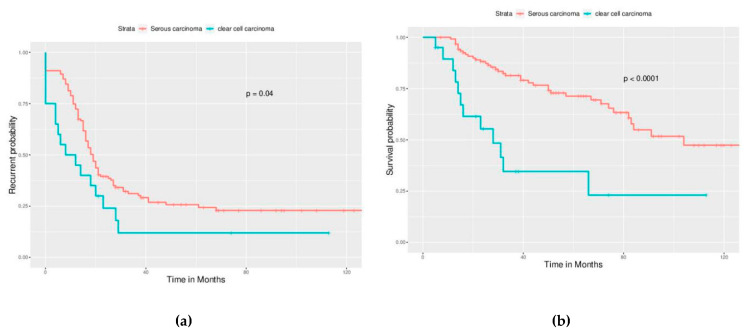
Survival curves by cell type (All enrolled cases), focus on serous type, and clear cell type. (**a**) PFS, *p* = 0.04, (**b**) OS, *p* < 0.0001. The outcome of clear cell carcinoma is worse than serous carcinoma.

**Figure 4:**
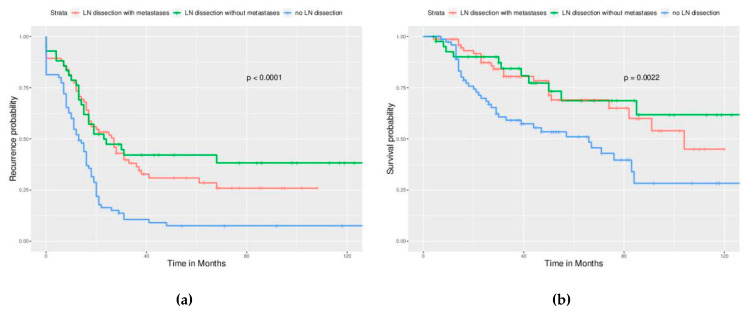
Survival curves by the LN status (All enrolled cases) (**a**) PFS, *p* < 0.0001, (**b**) OS, *p* = 0.0022. LN dissection provided better outcome of all enrolled cases, no matter how LN status was.

**Figure 5:**
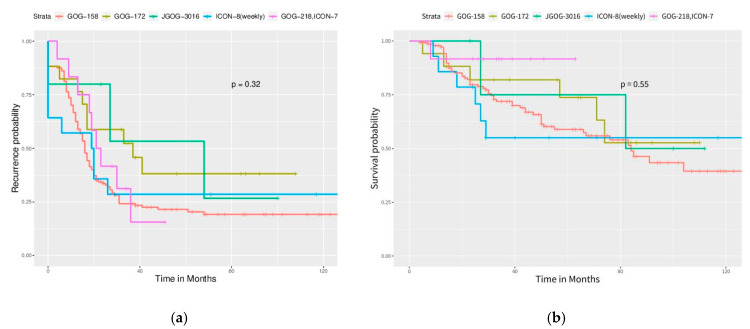
Survival curves by adjuvant chemotherapy regimens (All enrolled cases). However, no statistical significance is observed. (**a**) PFS, *p* = 0.32, (**b**) OS, *p* = 0.55.

Significant differences in PFS and OS could be observed in different FIGO stages (Figure 2). More advanced stages reduced PFS and OS as expected. Different cell types also caused significant differences in PFS and OS, especially serous and clear cell type (Figure 3). Survival analysis for LN dissection revealed worse PFS and OS when LN dissection was omitted. PFS and OS were comparable between cases with an LN metastasis status that is positive or negative (Figure 4). No significant differences were observed in PFS and OS between different adjuvant chemotherapies (Figure 5).

### 3.2. Analysis of Cases with Optimal Debulking Surgery

#### 3.2.1. Factors Affecting Disease Recurrence/Persistence and Survival

The FIGO stage (*p* = 0.023) and LN status (*p* = 0.015) still significantly affected recurrence, and cell types and adjuvant chemotherapies did not (Table 8). Furthermore, LN status (*p* = 0.01) significantly affected survival, but FIGO stage, cell types, and adjuvant chemotherapy did not (Table 9). Again, choice of adjuvant chemotherapy played little role in recurrence/persistence and survival even though optimal debulking was achieved.

#### 3.2.2. Risk Assessment

The cox regression hazard model (Table 10 and Table 11) confirmed that a more advanced FIGO stage increased risks of disease recurrence/persistence, and that stage IVB increased the risk of mortality. A clear cell type had a higher risk of disease recurrence/persistence (HR = 3.992, 95% CI = 1.563–10.196, *p* = 0.004) and had mortality (HR = 7.694, 95% CI = 2.266–26.124, *p* = 0.001) than the serous type. The endometroid type was also found to have a higher risk of mortality (HR = 13.153, 95% CI = 1.286–134.542, *p* = 0.03) than the serous type. LN dissection, whether negative or positive for metastasis, led to a lower risk of disease recurrence/persistence (HR = 0.149 and 0.173, respectively, 95% CI = 0.055–0.405 and 0.076–0.391, respectively, both *p* < 0.001) and mortality (HR = 0.052 and 0.226, respectively, 95% CI = 0.008–0.348 and 0.073–0.704, respectively, *p* = 0.002 and 0.01, respectively) than lack of LN dissection. None of the modified chemotherapy regimens demonstrated a higher or lower risk of disease recurrence/persistence than the traditional regimen from GOG-158. These results are similar to those in all enrolled cases. However, intraperitoneal chemotherapy from GOG-172 in an optimal debulking group reduced the risk of mortality when compared with chemotherapy from GOG-158 (HR = 0.233, 95% CI = 0.057–0.95, *p* = 0.0421).

#### 3.2.3. Survival Analysis

**Figure 6:**
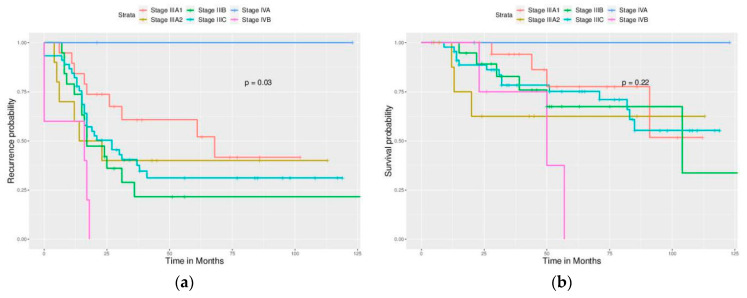
Survival curves based on the FIGO stage (Optimally debulked group). (**a**) PFS, *p* = 0.03. (**b**) OS, *p* = 0.22.

**Figure 7:**
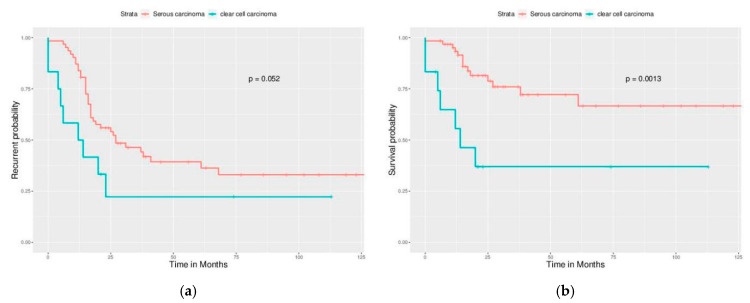
Survival curves by cell type (Optimally debulked group). Focus on serous type and clear cell type. Clearly, clear cell carcinoma still has a worse outcome than serous carcinoma. (**a**) PFS, *p* = 0.052, (**b**) OS, *p* = 0.00013.

**Figure 8:**
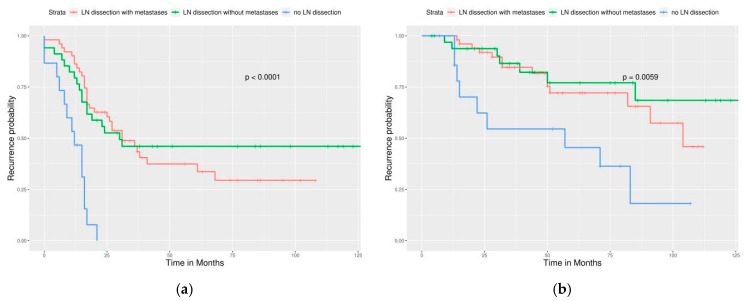
Survival curves by the LN status (Optimally debulked group) (**a**) PFS, *p* < 0.0001, (**b**) OS, *p* = 0.0059. LN dissection provided a significantly better outcome of cases, even in an optimally debulked group.

**Figure 9:**
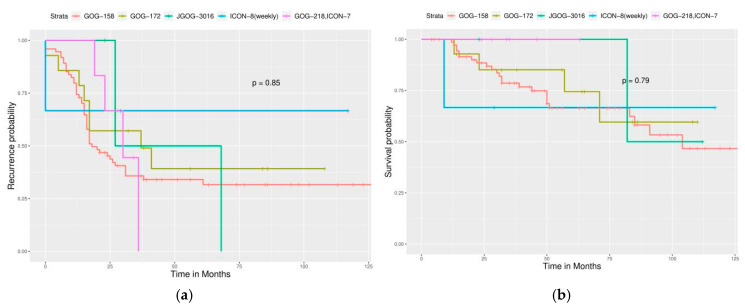
Survival curves by adjuvant chemotherapy regimens (Optimally debulked group) (**a**) PFS, *p* = 0.85, (**b**) OS, *p* = 0.79.

For different FIGO stages, significant differences were noted in PFS but not in OS (Figure 6). Different cell types still caused significant differences in PFS and OS (Figure 7). Survival analysis for LN dissection revealed worse PFS and OS when LN dissection was omitted, even in optimally debulked cases. PFS and OS were comparable between cases with an LN metastasis status that is positive or negative (Figure 8). Lastly, no significant differences were observed in PFS and OS between different adjuvant chemotherapies in this group (Figure 9). However, intraperitoneal chemotherapy might reduce mortality risk.

### 3.3. Analysis of Cases without an Optimal Debulking Surgery

**Figure 10:**
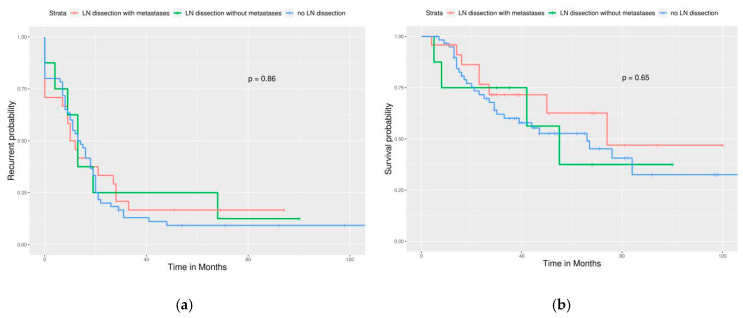
Survival curves of PFS and OS by LN status (Sub-optimally debulked group). (**a**) PFS, *p* = 0.86. (**b**) OS, *p* = 0.65. This means that LN dissection could be omitted if optimal debulking cannot be achieved.

LN dissection in this group failed to improve PFS and OS (Figure 10). This means that LN dissection can be omitted if optimal debulking cannot be achieved.

## 4. Discussion

In 2016, 1507 newly diagnosed EOC cases in Taiwan based on the cancer registry from Health Promotion Administration, the Ministry of Health and Welfare [13]. In general, approximately 70–75% of cases with EOC present at an advanced stage (≥IIIA). However, in our institute, only half of the cases with EOC had an advanced stage at the time of diagnosis. A high recurrence rate within two years after primary management and low five-year survival rate in advanced EOC are the same as those in Western countries [14].

Serous carcinoma is the most common histological type in EOC worldwide. A higher incidence of clear cell carcinoma is observed in East and South Asian countries than in Western countries [15,16]. Ovarian clear cell carcinoma is often diagnosed at an early stage. In our cases with advanced EOC, the clear cell type was the second most common cell type (10.4%). Shu et al. reported that the median OS for stage III/IV ovarian clear cell carcinoma was 37.0 and 28.7 months, respectively [17]. Chan et al. reported a worsened five-year survival rate for clear cell carcinoma than for serous carcinoma for patients with stage III (31.5% vs. 35.0%, *p* < 0.001) and stage IV (17.5% vs. 22.2%, *p* < 0.001) disease [18]. We observed similar results in our analysis. Clear cell carcinoma had a worse outcome than serous carcinoma, including a higher risk of recurrence/persistence, higher risk in mortality, and shorter PFS and OS even in cases with optimal debulking. The number of cases with other histological types were too few in our study for statistical analysis.

On the basis of the revised FIGO staging system (2014), LN metastasis is newly re-classified as stage IIIA1 instead of IIIC [3]. We, therefore, re-classified cases with LN metastases according to the new FIGO staging system. Stage IIIC was “macroscopic peritoneal metastases beyond the pelvis >2 cm in the greatest dimension regardless of the LN status.” We found that most our patients were stage IIIC (50%) and that patients with only retroperitoneal LN metastases without intraperitoneal lesions (re-classified stage IIIA1) had better PFS and OS than the re-classified IIIC cases. This finding supports the downstaging of the LN status to stage IIIA1 by FIGO.

The completeness of surgical debulking plays a critical role in treating advanced EOC. Optimally debulked cases in our study had significantly longer PFS and OS than those cases with suboptimal or no debulking. This finding is compatible with the current knowledge of treating EOC. Moreover, we found that the outcome between patients with suboptimal debulking and no debulking was similar. Thus, surgeons should do their best to achieve optimal debulking or abort debulking surgery if this goal cannot be achieved. Neoadjuvant chemotherapy and optimal interval debulking surgery could be considered to improve the outcome of such difficult cases [19]. In our study, cases with an optimal interval debulked surgery after neoadjuvant chemotherapy (data not shown) were classified into our optimally debulked group, and they also had better PFS and OS than the primary sub-optimally debulked group or no debulking group.

Retroperitoneal LN dissection is included in the debulking and staging procedure and improves the completeness of debulking surgery, but its benefits to patients’ outcomes is debatable. Benedetti et al. reported that systematic lymphadenectomy in optimally debulked EOC improves PFS, but not OS in their randomized trial [20]. However, the trial by Harter et al. in 2019 reported no improvement of PFS and OS if systematic retroperitoneal lymphadenectomy was performed in advanced EOC without residual intraperitoneal macroscopic lesions [21]. This study excluded cases with evident lymphadenopathy before preoperative imaging and demonstrated that additional LN dissection for non-bulky LNs was associated with frequent surgical complications.

Many gynecological oncologists choose to omit retroperitoneal LN dissection when peritoneal carcinomatosis is identified during surgery (stage ≥ IIIA2) if preoperative imaging does not reveal lymphadenopathy. This is because LN metastasis does not up-stage the disease. Furthermore, omitting LN dissection shortens the surgical time, and, thus, decreases morbidity. They think that microscopic LN metastases could likely be treated by adjuvant target chemotherapy [21]. On the basis of our findings, we recommend LN dissection in all cases undergoing optimal debulking surgery, even with no evidence of LN metastases in order to achieve better PFS and OS and a lower risk of recurrence/persistence and mortality. However, LN dissection was not found to improve survival in our sub-optimally debulked cases. Therefore, it could be omitted in such a scenario.

EOC is the most chemo-sensitive solid tumor. Therefore, adjuvant chemotherapy plays a critical role in its treatment [14]. Various regimens have been studied for decades, and the platinum-based combination has been the regimen of choice since 1990. The three-weekly regimen of intravenous paclitaxel and carboplatin/cisplatin after surgical debulking is the current standard of care since the consensus meeting of International Gynecological Cancer Intergroup Ovarian Cancer (GCIS) since 2005 [14]. Many clinical trials have tried various modifications for this regimen to improve patient outcomes [6,7,8,9] such as changing the route of administration (from intravenous to intraperitoneal), schedule of regimen (dose-dense weekly infusion), and adding newly developed target therapeutic agents (such as a tyrosine-kinase inhibitor or a monoclonal antibody against vascular epithelial growth factor). The milestones of these trials are summarized in Table 2. Although they revealed more promising results than the traditional regimen, a direct cross-trial comparison is unacceptable and unreasonable, which precludes the determination of the best new regimen. Many pros and cons are discussed in these published trials. For example, intraperitoneal chemotherapy (GOG-172) has a higher treatment-related toxicity and morbidity than a traditional intravenous regimen, and the completion rate of the total six courses is only 44%. Dose dense, weekly schedule of paclitaxel infusion (JGOG-3016 [7]) is the only prospective trial with such a promising result, but it was not reproduced by other identical schedule trials (GOG-262, MITO-7, and ICON-8) in Western countries [22]. ICON-8, which included one arm of weekly paclitaxel, three-weekly carboplatin, and one arm of weekly paclitaxel–carboplatin did not show improvement from dose-dense chemotherapy [10]. Adding bevacizumab (GOG-218/ICON-7) improved PFS but not OS in a recent long-term, follow-up trial [8].

The selection of the adjuvant chemotherapy regimen depends on many factors, such as surgical completeness, clinicians’ preference and experience, cases’ performance status, and economic status. In the present retrospective study, we tried to find the regimen with the best outcome in our daily practice. We found that no new or modified adjuvant chemotherapies led to a lower risk of recurrence/persistence or death or better PFS and OS than the traditional regimen from GOG-158, even when a sub-analysis of cases with optimal debulking was performed. Intraperitoneal chemotherapy (from GOG-172) in the optimally debulked group was the only regimen that showed a change including lower risk of mortality than the traditional regiment. However, no PFS or OS benefit was noted. Too few cases were administered using this regimen in our study, which likely contributed to this result. In addition, a high withdrawal rate was observed, which was the same as GOG-172, because of the intolerable side effects of this regimen. Grade 3–4 hematologic toxicities are frequently encountered on Day 8 with infusion of intraperitoneal paclitaxel. Currently, the application of cisplatin in first-line chemotherapy is lower than carboplatin. Carboplatin has lower renal toxicity, and it can be timesaving in paclitaxel infusion (from 24 h infusion to 3 h infusion) [5]. Therefore, this intraperitoneal regimen is not chosen as the first-line treatment by many clinicians, even if it demonstrated excellent improvement of PFS and OS in a clinical trial. It is possible that a higher number of cases in that arm in our study could have demonstrated a survival difference.

In the arm of chemotherapy plus bevacizumab, we did not have sufficient cases to analyze because we considered enrollment of cases until only December 2017. This was because we wanted to evaluate the actual recurrence rate because most cases with advanced EOC experience recurrence within 24 months [5,6,7,8,9,10]. In our institute, adding bevacizumab in chemotherapy became more frequent after 2017. Thus, there should be more cases from 2018, and they could be enrolled in a future study. For economic consideration, the dose of bevacizumab is 7.5 mg/kg in our daily practice, which is the same as ICON-7 [9].

Our study has some limitations. Its retrospective design results in a lower strength of evidence than prospective studies. Non-balanced case numbers in different groups of adjuvant chemotherapies (75% of cases received the traditional regimen of GOG-158) likely resulted in a statistical bias. Lack of precise surgical records in ancient cases undergoing optimal debulking surgery (no macro-residual, <1 or <2 cm residual) increased the heterogenicity of the optimally debulked group. The scale of LN dissection (biopsy only, regional sampling, or systemic lymphadenectomy) was sometimes difficult to determine from ancient surgical records. A larger multi-center retrospective study might be considered and conducted in the future by the Taiwanese Gynecological Oncology Study Group to collect more experience from the real world.

## 5. Conclusions

Optimal debulking surgery remains a critical part of treating advanced EOC. Retroperitoneal LN dissection should always be performed for optimally debulked cases to reduce the risk of recurrence/persistence and death and to improve survival. However, LN dissection can be omitted when optimal debulking cannot be achieved.

On the basis of our limited data, different new settings of chemotherapies (such as adding bevacizumab and maintenance) demonstrated only comparable PFS, OS, and recurrence/persistence risk, irrespective of the achievement of optimal debulking. Thus, the traditional three-weekly intravenous paclitaxel–carboplatin remains the first choice of chemotherapy. Lastly, clear cell EOC, which is more frequent in East and South Asia, might have worse outcomes than serous EOC, which requires aggressive treatment and follow-up.

## Figures and Tables

**Table 1:** Basic characteristics.

n = 192	
Median age at diagnosis (years old), (range)	53 ± 10.57 (29–81)
Body weight (kg)	57 ± 10.18 (36–98)
Body mass index (BMI, kg/m^2^)	23.0 ± 4.23 (15.8–40.3)
**FIGO (Federation of Gynecology and Obstetrics) stage (2014)**	
IIIA1	19 (9.9%)
IIIA2	10 (5.2%)
IIIB	21 (10.9%)
IIIC	100 (52.1%)
IVA	13 (6.8%)
IVB	29 (15.1%)
**Cell type**	
Serous	123 (64.1%)
Mucinous	8 (4.2%)
Seromucinous	2 (1.0%)
Clear cell	20 (10.4%)
Endometrioid	12 (6.3%)
Brenner (including transitional cell carcinoma (TCC))	3 (1.6%)
Mixed type	15 (7.8%)
Undifferentiated	8 (4.2%)
Squamous cell	1 (0.5%)
**Surgical completeness**	
Optimal debulking surgery	100 (52.1%)
Sub-optimal debulking surgery	75 (39.1%)
Biopsy or only cytology proof	17 (8.9%)
**Lymph nodes metastases**	
Positive of metastases (pathology proof)	75 (39.1%)
Negative of metastases (pathology proof)	42 (21.9%)
No lymph nodes dissection or unknown	75 (39.1%)
**Adjuvant chemotherapy (for details, please refer to Table 2)**	
GOG-158	144 (75.0%)
GOG-172	17 (8.9%)
JGOG-3016	5 (2.6%)
ICON-8	14 (7.3%)
GOG-218/ICON-7	12 (6.3%)
**Recurrent status**	
No	48 (25.0%)
Yes	117 (60.9%)
Persistent disease (no response to first line chemotherapy)	27 (14.1%)
**Survival status**	
No evidence of disease (NED)	47 (24.5%)
Alive with disease (AWD)	74 (38.5%)
Died of disease (DOD)	71 (37.0%)
**Survival interval (months)**	
Progression-free survival (PFS)	17 ± 30.96 (0–167)
Overall survival (OS)	39 ± 32.54 (4–167)

**Table 2:** Summary of milestones of adjuvant chemotherapy based on prior clinical trials.

Group		Regimen (Listed Test-Arm Only)	Published	PFS ^1^	OS ^1^	Reference
1	GOG-158	Three-weekly carboplatin AUC five and three-weekly paclitaxel 175 mg/m^2^	2003	20.7 vs. 19.4	57.4 vs. 48.7	[5]
2	GOG-172	Three-weekly IV paclitaxel 135 mg/m^2^ (24-h) and IP cisplatin 100 mg/m^2^ on day 2, IP paclitaxel 60 mg/m^2^ on day 8	2007	23.8 vs. 18.3	65.6 vs. 49.7	[6]
3	JGOG-3016	Three-weekly carboplatin AUC5 and weekly paclitaxel 80 mg/m^2^	2013	28.2 vs. 17.5	100.5 vs. 62.2	[7]
4	ICON-8	weekly carboplatin AUC2 and weekly paclitaxel 80 mg/m^2^	2019	21.0 vs. 20.8 vs. 17.7	N/A ^2^	[10]
5	GOG-218ICON-7	Three-weekly carboplatin AUC 5 and three-weekly paclitaxel 175 mg/m^2^ with three-weekly bevacizumab (15 or 7·5 mg/kg)	2011 and 2015	18.0 vs. 12.0 (GOG-218) 19.0 vs. 17.3 (ICON-7)	43.4 vs. 40.8 vs. 41.1 (GOG-218) 39.3 vs. 34.5 (ICON-7)	[8,9]

^1^ PFS and OS (months) were compared with the control arm(s) in the prior clinical trials. ^2^ Not available.

**Table 3:** Case summaries according to surgical completeness.

Parameters	Surgical Completeness (Cases, n = 192)			
	Optimal Debulking Surgery	Sub-Optimal Debulking Surgery	Biopsy or Only Cytology Proof	*p*-Value
Mean age at diagnosis (years-old) ^$^	52.79 ± 10.42	56.79 ± 10.85	51.88 ± 8.20	0.043 *^,1^
Body weight (kg) ^$^	57.08 ± 10.16	58.13 ± 10.38	61.94 ± 8.80	0.233 ^1^
Body mass index (BMI, kg/m^2^) ^$^	23.63 ± 4.21	23.80 ± 4.23	25.42 ± 4.28	0.441 ^1^
Progression free survival (PFS) ^#^	22 ± 32.07	12 ± 30.18	18 ± 19.12	<0.001 *^,2^
Overall survival (OS) ^#^	47.5 ± 33.03	33 ± 32.68	29 ± 24.40	<0.001 *^,2^

^1^ Student’s t test. * Statistical significance. ^2^ Log-rank test. ^$^ Expressed as mean ± standard deviation. ^#^ Expressed as median ± standard deviation.

**Table 4:** Parameters affect recurrent/persistent status in all enrolled cases.

Parameters	Recurrent Status (Cases, n = 192)			
	No	Yes	Persistent	*p*-Value ^1^
**FIGO stage (2014)**				0.001 *
IIIA1	10	9	0	
IIIA2	4	5	1	
IIIB	6	15	0	
IIIC	24	64	12	
IVA	2	8	3	
IVB	2	16	11	
**Cell type**				0.444
Serous	33	79	11	
Mucinous	2	3	3	
Sero-mucinous	0	2	0	
Clear cell	3	11	6	
Endometrioid	4	6	2	
Brenner (including TCC)	1	2	0	
Mixed type	3	8	4	
Undifferentiated	2	5	1	
Squamous cell	0	1	0	
**Surgical completeness**				<0.001 *
Optimal debulking surgery	37	57	6	
Sub-optimal debulking surgery	9	50	16	
Biopsy or only cytology proof	2	10	5	
**Lymph nodes metastases**				<0.001 *
Positive of metastases (pathology proof)	24	43	8	
Negative of metastases (pathology proof)	17	21	4	
No lymph nodes dissection or unknown	7	53	15	
**Adjuvant chemotherapy (For details, please refer to Table 2)**				0.10
GOG-158	32	94	18	
GOG-172	7	7	3	
JGOG-3016	2	2	1	
ICON-8	4	5	5	
GOG-218/ICON-7	3	9	0	

^1^ Chi-square test or Fisher’s exact test. * Statistical significance.

**Table 5:** Parameters affect survival status in all enrolled cases.

Parameters	Survival Status (Cases, n = 192)			
	NED ^2^	AWD ^3^	DOD ^4^	*p*-Value ^1^
**FIGO stage (2014)**				0.062
IIIA1	10	5	4	
IIIA2	4	3	3	
IIIB	5	10	6	
IIIC	23	42	35	
IVA	2	4	7	
IVB	3	10	16	
**Cell type**				0.333
Serous	31	54	38	
Mucinous	2	2	4	
Sero-mucinous	0	2	0	
Clear cell	3	5	12	
Endometrioid	5	2	5	
Brenner (including TCC)	1	1	1	
Mixed type	3	4	8	
Undifferentiated	2	4	2	
Squamous cell	0	0	1	
**Surgical completeness**				0.004 *
Optimal debulking surgery	36	34	30	
Sub-optimal debulking surgery	9	33	33	
Biopsy or only cytology proof	2	7	8	
**Lymph nodes metastases**				<0.001 *
Positive of metastases (pathology proof)	23	30	22	
Negative of metastases (pathology proof)	18	13	11	
No lymph nodes dissection or unknown	6	31	38	
**Adjuvant chemotherapy (For details, please refer to Table 2)**				0.244
GOG-218/ICON-7	31	57	16	
GOG-172	7	4	6	
JGOG-3016	2	1	2	
ICON-8	4	4	6	
GOG-218/ICON-7	3	8	1	

^1^ Chi-square test or Fisher’s exact test. ^2^ NED: No evidence of disease. ^3^ AWD: Alive with disease. ^4^ DOD: Death of disease. * Statistical significance.

**Table 6:** Cox regression hazard model for recurrence/persistence risk in all enrolled cases.

Factor(s)	HR ^1^	95% CI ^2^	*p*-Value
**FIGO stage**: IIIA1	1 ^#^		
IIIA2	1.985	0.605–6.514	0.258
IIIB	1.831	0.729–4.595	0.198
IIIC	2.280	1.042–4.989	0.039 *
IVA	3.782	1.372–10.427	0.010 *
IVB	3.864	1.588–9.401	0.003 *
**Cell types**: serous	1 ^#^		
mucinous	1.035	0.424–2.524	0.940
Clear cell	2.452	1.377–4.365	0.002 *
Endometrioid	1.433	0.653–3.145	0.369
Mixed type	1.714	0.916–3.207	0.092
**Lymph nodes metastases**: No lymph nodes dissection or unknown	1 ^#^		
Positive of metastases (pathology proof)	0.528	0.331–0.845	0.008 *
Negative of metastases (pathology proof)	0.331	0.162–0.585	<0.001 *
**Adjuvant chemotherapy**: GOG-158	1 ^#^		
GOG-172	0.605	0.302–1.211	0.605
JGOG-3016	0.922	0.280–3.036	0.922
ICON-8	0.646	0.325–1.283	0.646
GOG-218/ICON-7	0.794	0.392–1.608	0.794

^1^ HR: Hazard ratio. ^2^ CI: confident interval. ^#^ Reference item. * *p* < 0.05.

**Table 7:** Cox regression hazard model for mortality risk in all enrolled cases.

Factor(s)	HR ^1^	95% CI ^2^	*p* Value
**FIGO stage**: IIIA1	1 ^#^		
IIIA2	2.222	0.383–12.882	0.373
IIIB	1.289	0.293–5.677	0.737
IIIC	2.049	0.618–6.793	0.241
IVA	6.286	1.442–27.404	0.014 *
IVB	5.311	1.420–19.856	0.013 *
**Cell types**: serous	1 ^#^		
mucinous	2.142	0.693–6.617	0.186
Clear cell	6.287	2.945–13.432	<0.001 *
Endometrioid	4.695	1.620–13.610	0.004 *
Mixed type	2.588	1.156–5.793	0.021 *
**Lymph nodes metastases**: No lymph nodes dissection or unknown	1 ^#^		
Positive of metastases (pathology proof)	0.559	0.287–1.088	0.087
Negative of metastases (pathology proof)	0.280	0.116–0.678	0.005 *
**Adjuvant chemotherapy**: GOG-158	1 ^#^		
GOG-172	0.752	0.288–1.967	0.562
JGOG-3016	1.499	0.331–6.786	0.599
ICON-8	0.720	0.288–1.800	0.482
GOG-218/ICON-7	0.271	0.037–2.008	0.201

^1^ HR: Hazard ratio. ^2^ CI: confident interval. ^#^ Reference item. * *p* < 0.05.

**Table 8:** Parameters affecting recurrence/persistence in optimally debulked cases.

Parameters	Recurrent Status (Cases, n = 100)			
	No	Yes	Persistent	*p*-Value ^1^
**FIGO stage (2014)**				0.023 *
IIIA1	10	9	0	
IIIA2	4	5	1	
IIIB	5	14	0	
IIIC	16	26	3	
IVA	2	0	0	
IVB	0	3	2	
**Cell type**				0.300
Serous	25	36	1	
Mucinous	1	2	0	
Sero-mucinous	0	1	0	
Clear cell	3	6	3	
Endometrioid	3	4	2	
Brenner (including TCC ^1^)	1	2	0	
Mixed type	3	5	0	
Undifferentiated	1	0	0	
Squamous cell	0	1	0	
**Lymph nodes metastases**				0.015 *
Positive of metastases (pathology proof)	20	30	1	
Negative of metastases (pathology proof)	16	16	2	
No lymph nodes dissection or unknown	1	11	3	
**Adjuvant chemotherapy (For more details, refer to Table 2)**				0.277
GOG-158	26	45	3	
GOG-172	6	6	2	
JGOG-3016	1	2	0	
ICON-8	2	0	1	
GOG-218/ICON-7	2	4	0	

^1^ Chi-square test or Fisher’s exact test. * Statistical significance.

**Table 9:** Parameters affecting survival in optimally debulked cases.

Parameters	Survivorship (Cases, n = 100)			
	NED	AWD	DOD	*p*-Value ^1^
**FIGO stage (2014)**				0.404
IIIA1	10	5	4	
IIIA2	4	3	3	
IIIB	4	9	6	
IIIC	15	16	14	
IVA	2	0	0	
IVB	1	1	3	
**Cell type**				0.475
Serous	23	25	14	
Mucinous	1	0	2	
Sero-mucinous	0	1	0	
Clear cell	3	2	7	
Endometrioid	4	2	3	
Brenner (including TCC)	1	1	1	
Mixed type	3	3	2	
Undifferentiated	1	0	0	
Squamous cell	0	0	1	
**Lymph nodes’ metastases**				0.010 *
Positive of metastases (pathology proof)	19	18	14	
Negative of metastases (pathology proof)	17	10	7	
No lymph nodes dissection or unknown	0	6	9	
**Adjuvant chemotherapy (For details, refer to Table 2)**				0.637
GOG-158	25	25	24	
GOG-172	6	4	4	
JGOG-3016	1	1	1	
ICON-8	2	0	1	
GOG-218/ICON-7	2	4	0	

^1^ Chi-square test or Fisher’s exact test. * Statistical significance.

**Table 10:** Cox regression hazard model for disease recurrence/persistence in optimally debulked cases.

Factor(s)	HR ^1^	95% CI ^2^	*p*-Value
**FIGO stage**: IIIA1	1 ^#^		
IIIA2	1.453	0.364–5.801	0.597
IIIB	2.774	0.905–8.504	0.074
IIIC	1.869	0.745–4.686	0.182
IVA	0.000	0.000	0.977
IVB	5.129	1.350–19.481	0.016 *
**Cell types**: serous	1 ^#^		
Mucinous	1.147	0.218–6.038	0.940
Clear cell	3.992	1.563–10.196	0.004 *
Endometrioid	1.165	0.368–3.685	0.795
Mixed type	1.221	0.436–3.416	0.704
**Lymph nodes metastases**: No lymph nodes dissection or unknown	1 ^#^		
Positive of metastases (pathology proof)	0.173	0.076–0.391	<0.001 *
Negative of metastases (pathology proof)	0.149	0.055–0.405	<0.001 *
**Adjuvant chemotherapy**: GOG-158	1 ^#^		
GOG-172	0.497	0.209–1.183	0.114
JGOG-3016	1.133	0.252–5.085	0.871
ICON-8	0.692	0.075–6.411	0.746
GOG-218/ICON-7	0.987	0.306–3.191	0.983

^1^ HR: Hazard ratio. ^2^ CI: confidence interval. ^#^ Reference item. * *p* < 0.05.

**Table 11:** Cox regression hazard model for mortality in optimally debulked cases.

Factor(s)	HR ^1^	95% CI ^2^	*p*-Value
**FIGO stage**: IIIA1	1 ^#^		
IIIA2	3.549	0.337–37.387	0.299
IIIB	2.097	0.313–14.052	0.445
IIIC	4.369	0.895–22.127	0.076
IVA	0.000	0.000	0.993
IVB	9.552	1.147–79.550	0.037 *
**Cell types**: serous	1 ^#^		
Mucinous	5.663	0.886–36.173	0.067
Clear cell	7.694	2.266–26.124	0.001 *
Endometrioid	13.156	1.286–134.542	0.030 *
Mixed type	0.512	0.080–3.277	0.480
**Lymph nodes metastases**: No lymph nodes dissection or unknown	1 ^#^		
Positive of metastases (pathology proof)	0.226	0.073–0.704	0.010 *
Negative of metastases (pathology proof)	0.052	0.008–0.348	0.002 *
**Adjuvant chemotherapy**: GOG-158	1 ^#^		
GOG-172	0.233	0.057–0.951	0.042 *
JGOG-3016	2.198	0.243–19.884	0.483
ICON-8	14.267	0.836–243.549	0.066
GOG-218/ICON-7	0.000	0.000	0.985

^1^ HR: Hazard ratio. ^2^ CI: confidence interval. ^#^ Reference item. * *p* < 0.05.
